# Gold Nanoparticles Embedded in PEDOT:PSS for Enhanced Hole Injection and Negligible Exciton Quenching in OLEDs

**DOI:** 10.3390/nano16140840

**Published:** 2026-07-08

**Authors:** Yijun Ning, Suling Zhao, Yingzhuang Ma, Xin Guo

**Affiliations:** 1 School of Optical and Electronic Information, Suzhou City University, Suzhou 215104, China; ningyijun458@163.com (Y.N.); yzhma@szcu.edu.cn (Y.M.); 2 Key Laboratory of Luminescence and Optical Information, Beijing Jiaotong University, Ministry of Education, Beijing 100044, China; slzhao@bjtu.edu.cn

**Keywords:** OLEDs, AuNPs, PEDOT:PSS, hole injection, exciton quenching

## Abstract

Gold nanoparticles (AuNPs) can improve hole injection in OLEDs but may also induce LSPR-related optical perturbation and exciton quenching. To exploit their hole-injection-promoting effect while suppressing possible exciton quenching, AuNPs with diameters of 3.5–4.0 nm were synthesized by citrate reduction and doped into PEDOT:PSS as a hole injection layer modifier. Photoluminescence measurements revealed absorption-related wavelength-dependent optical perturbation from exposed AuNPs, with the strongest effect on red emission and only a minor effect on blue emission. AuNPs embedded in PEDOT:PSS caused only slight changes in PL spectra and PL decay behavior, indicating that obvious exciton quenching was avoided. OLEDs with AuNP-doped PEDOT:PSS exhibited reduced turn-on voltages and increased maximum current efficiencies from 22.4 to 27.0 cd/A, 7.64 to 15.6 cd/A, and 16.8 to 29.0 cd/A for blue, red, and green devices, respectively. These results indicate that sub-5 nm AuNPs embedded in PEDOT:PSS can improve device performance by enhancing hole-injection capability, while their wavelength-dependent exciton-quenching effect is negligible.

## 1. Introduction

PEDOT:PSS has been widely employed as a hole-injection layer in solution-processed organic light-emitting diodes (OLEDs) to facilitate hole injection from ITO anodes. Nevertheless, hole injection across the ITO/PEDOT:PSS/organic interface remains imperfect because the electrical conductivity, work-function alignment, and interfacial properties of PEDOT:PSS are highly sensitive to processing conditions. Recent studies have shown that the electrical properties of nanoscale PEDOT:PSS layers can be effectively modulated by structural and chemical strategies, including multilayer deposition, acid treatment, graphene incorporation, and metal nanoparticle doping, thereby improving charge injection and transport in solution-processed optoelectronic devices [1].

Among various modifiers, gold nanoparticles (AuNPs) are particularly attractive because of their high electrical conductivity and their ability to regulate interfacial hole injection [2,3,4,5]. Justin et al. deposited AuNPs onto ITO anodes and, by optimizing the surface coverage of AuNPs relative to the hole injection layer, modulated the anode work function and significantly improved hole injection [6,7]; D. Wang et al. reported that sparsely distributed AuNPs on ITO enhanced hole injection and reduced OLED driving voltage via nanoparticle-induced local electric fields [8]. Tholkappiyan et al. found that attaching AuNPs onto n-type silicon (n-Si) substrates could enhance charge transport by promoting quantum tunneling [9]. Hwang et al. fabricated electrodes by attaching AuNPs to PEDOT molecules via vapor-phase deposition, which markedly increased the electrical conductivity of PEDOT [10]. In addition to interfacial modification of ITO, embedding metal nanostructures directly into PEDOT:PSS has emerged as another strategy to modify the hole-injection/transport layer. For example, Ag nanoparticle-based core–shell structures incorporated into PEDOT:PSS improved OLED performance through enhanced conductivity, improved hole injection, localized surface plasmon resonance, and possible light scattering [11]. These studies indicate that embedding metal nanostructures into PEDOT:PSS is a feasible strategy for optimizing OLED performance.

Nevertheless, metal nanostructures in OLEDs do more than facilitate charge injection. By modifying optical modes, surface-plasmon coupling, and waveguide-related losses [12], they may strongly influence exciton dynamics when positioned close to the emitting layer (EML), leading to either beneficial optical modulation or undesirable exciton quenching. In particular, when AuNPs are in direct contact with the emitting layer, severe exciton quenching may occur, thereby deteriorating device performance [13,14]. Kim et al. enhanced hole injection by doping 12 nm AuNPs into the hole injection layer; however, the luminescence performance of the resulting devices remained poor due to AuNP-induced quenching of emissive excitons [15]. This quenching is commonly explained by the fact that the LUMO level of the emitting layer is higher than the Fermi level of AuNPs. When the spatial separation between the two is sufficiently small, charge dissociation from emissive molecules can occur, leading to exciton quenching. Therefore, a thick blocking layer must generally be inserted between AuNPs and the emitting layer to eliminate the quenching effect, which is unfavorable for solution-processed devices.

To reduce the quenching effect of AuNPs on nearby excitons, numerous studies have employed surface ligands attached to AuNPs to increase the separation distance between AuNPs and excitons, thereby suppressing exciton quenching [16,17,18]. However, because ligand attachment is often unstable, this strategy is not well suited for high-performance light-emitting devices.

The electrical transport properties of AuNPs are beneficial for light-emitting devices; however, their adverse effect on emission quenching must be overcome. Therefore, balancing the hole-transport/injection-promoting function of AuNPs with their exciton-quenching effect represents an important issue worthy of investigation. The quenching of organic emissive molecules depends on multiple factors, including the spectral overlap between the emission wavelength and the surface plasmon energy of the nanoparticles, the size and morphology of AuNPs, and the separation distance between the emitting layer and AuNPs. Sub-5 nm AuNPs have a limited effective interaction range and are therefore expected to exert a weaker quenching effect on the emitting layer.

Since the pioneering work of Turkevich et al., solution-based chemical reduction has been widely used for the synthesis of AuNPs with controllable sizes and morphologies [19,20,21]. Subsequent studies further refined this strategy by adjusting key reaction parameters, such as the citrate-to-gold ratio, ligand composition, and solution pH, enabling the preparation of AuNPs over a broad size range [20,22,23]. Among these methods, citrate-mediated reduction of HAuCl_4_ remains a simple and practical route for preparing colloidal AuNPs with good dispersion [24,25]. In this work, sub-5 nm AuNPs with an average diameter of approximately 4 nm were synthesized by citrate reduction and incorporated into PEDOT:PSS as hole-injection modifiers. Red, green, and blue OLEDs were fabricated to evaluate the influence of AuNP-modified PEDOT:PSS on hole injection, exciton quenching, and device performance across different emission wavelengths.

## 2. Materials and Methods

### 2.1. Materials

#### 2.1.1. Citrate Reduction Synthesis of Small-Sized AuNPs

Small-sized AuNPs were prepared via citrate reduction of HAuCl_4_ in aqueous solution. In brief, 0.2 mL of HAuCl_4_ solution (5 × 10^−2^ mol/L) was added to 100 mL of ultrapure water and heated to 100 °C with magnetic stirring. Then, 2 mL of trisodium citrate solution (5 × 10^−2^ mol/L) was added, followed by an additional 1.5 mL after 15 min. The mixture was stirred at 100 °C for 1 h until a wine-red AuNP colloidal solution was formed and then cooled to room temperature under continuous stirring.

#### 2.1.2. Materials for Emitting Layers and Charge-Transport Layers

High-performance small-molecule host–guest systems were employed for the red, green, and blue emitting layers, namely 15 wt% Ir(MDQ)_2_acac: mCBP, 15 wt% 4CzIPN: mCP, and 15 wt% DMAC-DPS: mCP, respectively. PEDOT:PSS, poly(3,4-ethylenedioxythiophene):poly(styrene sulfonate), was used as the hole injection material, and Clevios P AI 4083 was employed in this work.

The red-emitting guest Ir(MDQ)_2_acac, bis(2-methyldibenzo[f,h]quinoxaline)(acetylacetonate)iridium(III), the blue-emitting guest DMAC-DPS, 10,10′-(4,4′-sulfonylbis(4,1-phenylene))bis(9,9-dimethyl-9,10-dihydroacridine), and the electron-transport material POT2T, 2,4,6-tris[3-(diphenylphosphinyl)phenyl]-1,3,5-triazine, were purchased from Lumtec Luminescence Technology Corp., Hsinchu, Taiwan. The green-emitting guest 4CzIPN, 2,4,5,6-tetrakis(carbazol-9-yl)-1,3-dicyanobenzene, the host materials mCBP, 3,3′-di(9H-carbazol-9-yl)biphenyl, and mCP, 1,3-di-9-carbazolylbenzene, as well as the electron-transport materials DPEPO, bis[2-(diphenylphosphino)phenyl] ether oxide, and TPBi, 2,2′,2″-(1,3,5-benzinetriyl)-tris(1-phenyl-1H-benzimidazole), were purchased from Xi’an Yuri Solar Co., Ltd., Xi’an, China. All materials were used as received without further purification.

### 2.2. Methods

Indium tin oxide (ITO) glass substrates were sequentially ultrasonically cleaned with acetone, absolute ethanol, and deionized water. After being dried with nitrogen, the substrates were treated in a plasma cleaner for 90 s for surface cleaning. The PEDOT:PSS aqueous solution was filtered through a 0.45 μm filter and then mixed with the AuNP aqueous solution at different ratios. The mixed solutions were stirred on a magnetic stirring platform for 2 h to obtain uniformly AuNP-doped PEDOT:PSS aqueous solutions. PEDOT:PSS solutions containing different concentrations of AuNPs were spin-coated onto ITO substrates in air at 4000 rpm, followed by annealing on a hot plate at 150 °C for 15 min. Subsequently, the remaining functional layers were prepared in a nitrogen-filled glovebox.

For the fabrication of blue OLEDs, DMAC-DPS and mCP were separately dissolved in chlorobenzene to prepare clear solutions with a concentration of 10 mg/mL. The two solutions were mixed at a mass ratio of 1:0.15 and stirred for 4 h. The resulting emitting-layer solution was spin-coated onto the PEDOT:PSS substrate at 2000 rpm for 45 s, followed by annealing on a hot plate at 60 °C for 25 min. Finally, DPEPO, TPBi, LiF, and Al films were deposited by thermal evaporation under a vacuum pressure of 5 × 10^−4^ Pa at an evaporation rate of 0.5 Å/s.

For red OLEDs, the phosphorescent material Ir(MDQ)_2_acac, which exhibits high emission intensity, was selected as the red-emitting guest. The fabrication procedure of the red OLEDs was designed based on reported device structures, host–guest ratios, and solution concentrations. Ir(MDQ)_2_acac and mCBP were separately dissolved in chlorobenzene to prepare clear solutions with a concentration of 10 mg/mL. The two solutions were mixed at a mass ratio of 1:0.15 and stirred for 4 h. The mixed emitting-layer solution was spin-coated onto the PEDOT:PSS substrate at 2000 rpm for 45 s, followed by annealing on a hot plate at 80 °C for 20 min. Finally, POT2T, LiF, and Al films were deposited by thermal evaporation under a vacuum pressure of 5 × 10^−4^ Pa at an evaporation rate of 0.5 Å/s.

For green OLEDs, 4CzIPN, a commonly used high-efficiency emissive molecule, was selected as the green-emitting guest. The fabrication procedure of the green OLEDs was designed based on reported device structures, host–guest ratios, and solution concentrations. 4CzIPN and mCP were separately dissolved in chlorobenzene to prepare clear solutions with a concentration of 10 mg/mL. The two solutions were mixed at a mass ratio of 1:0.15 and stirred for 4 h. The resulting emitting-layer solution was spin-coated onto the PEDOT:PSS substrate at 2000 rpm for 45 s, followed by annealing on a hot plate at 60 °C for 25 min. Finally, DPEPO, TPBi, LiF, and Al films were deposited by thermal evaporation under a vacuum pressure of 5 × 10^−4^ Pa at an evaporation rate of 0.5 Å/s.

## 3. Results and Discussion

### 3.1. Preparation and Optical Characterization of Small-Sized AuNPs

**Figure 1:**
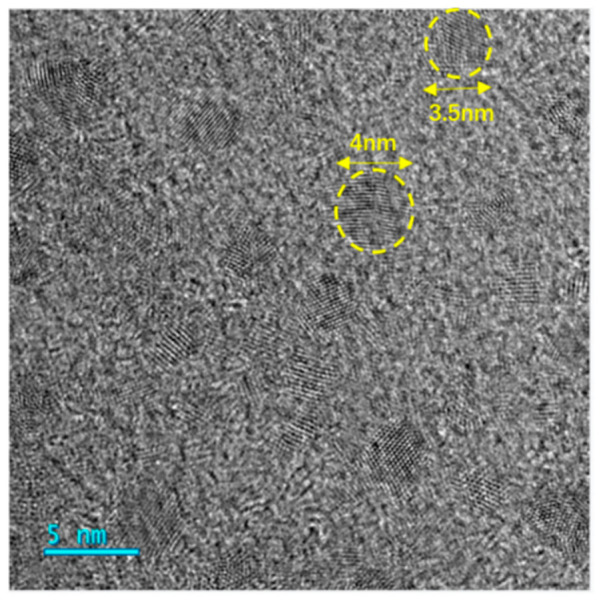
TEM image of the synthesized small-sized AuNPs.

Uniform particle size is critical for maintaining the stable properties of AuNPs. In this work, a relatively low concentration of gold precursor was used, leading to the formation of uniform small-sized AuNPs. Figure 1 shows the transmission electron microscopy (TEM) image of the synthesized AuNPs. The particles exhibit diameters of approximately 3.5–4.0 nm, with a narrow size distribution and good dispersion, making them suitable for incorporation into organic light-emitting devices.

**Figure 2:**
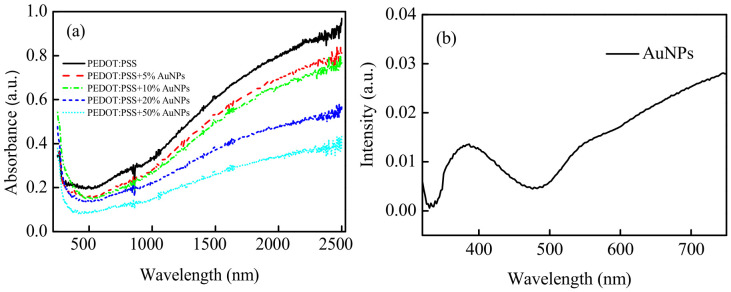
Absorption spectra of (**a**) PEDOT:PSS films doped with different concentrations of AuNPs and (**b**) the AuNP film deposited on a quartz substrate.

Figure 2a shows the absorption spectra of PEDOT:PSS films before and after AuNP doping. It should be noted that the AuNP doping ratios in this work refer to the volume ratios of the AuNP aqueous dispersion added to the PEDOT:PSS aqueous solution before spin coating. Pristine PEDOT:PSS exhibits a weak absorption peak centered at approximately 850 nm, together with a broad absorption band extending into the near-infrared region. After AuNP doping, the overall absorption intensity of the films gradually decreases with increasing doping ratio over the measured spectral range. This decrease may be related to changes in the PEDOT:PSS solution concentration and film formation behavior after introducing the AuNP aqueous solution. Meanwhile, small-sized AuNPs covered by PEDOT:PSS do not introduce pronounced additional optical absorption into the films, indicating limited optical perturbation in the PEDOT:PSS-embedded state.

The absorption spectrum of the pristine AuNP film deposited on a quartz substrate was further measured, as shown in Figure 2b. The AuNPs/quartz sample was prepared mainly to evaluate the wavelength-dependent absorption behavior of bare solid-state AuNPs. The weak absorbance of approximately 0.01 suggests a limited optical contribution from the low-coverage AuNP layer, while the broad absorption feature may arise from particle-size distribution and local heterogeneity. Since AuNPs are embedded in the PEDOT:PSS of OLED, the AuNPs/quartz sample should be regarded only as a reference for the optical response of bare AuNPs.

Specifically, the AuNPs/quartz sample shows a distinct absorption peak at approximately 386 nm and a weak absorption feature near 550 nm, while the absorption intensity gradually increases from 470 to 750 nm. These results indicate that the bare AuNPs prepared in this work exhibit weak absorption near the blue-emission wavelength, whereas their absorption gradually increases toward the green and red spectral regions. This spectral feature may also partly explain why many AuNP-related LSPR studies have focused mainly on red and green emission.

### 3.2. Influence of AuNPs on the PL Characteristics of Emissive Materials with Different Emission Wavelengths

Based on the wavelength-dependent absorption characteristics of the AuNP film discussed above, the influence of small-sized AuNPs on the PL spectra and decay dynamics of EMLs with different emission wavelengths was further investigated.

The PL spectra of host–guest-doped red, green, and blue emitting layers were measured on PEDOT:PSS, AuNP-doped PEDOT:PSS, and pristine AuNPs substrates, respectively. High-performance small-molecule host–guest systems were employed for the red, green, and blue emitting layers, namely 15 wt% Ir(MDQ)_2_acac: mCBP, 15 wt% 4CzIPN: mCP, and 15 wt% DMAC-DPS: mCP, respectively.

The test structures were quartz/PEDOT:PSS/EML (red, green, and blue), quartz/PEDOT:PSS + AuNPs/EML (red, green, and blue), and quartz/AuNPs/EML (red, green, and blue).

**Figure 3:**
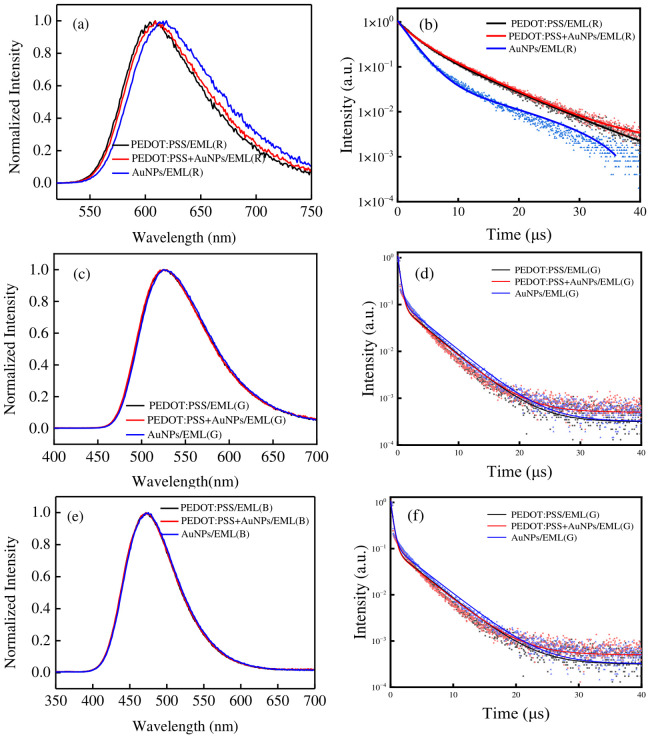
Normalized PL spectra and normalized PL decay curves measured at the emission peaks of red, green, and blue EMLs on different substrates: (**a**,**b**) red EML; (**c**,**d**) green EML; and (**e**,**f**) blue EML.

Figure 3 shows the PL spectra and normalized PL decay curves measured at the emission peaks of the red, green, and blue emissive materials on different substrates. As shown in Figure 3a,c,e, the influence of AuNPs on the PL spectra of the emitting layers exhibits wavelength dependence. For the red emitting layer, the effect of AuNPs is the most pronounced. When AuNP-doped PEDOT:PSS is used as the underlying layer, the PL spectrum shows only a slight red shift compared with that on pristine PEDOT:PSS. In contrast, when the emitting layer is directly spin-coated onto the pristine AuNP film, a more obvious red shift and long-wavelength broadening are observed. This behavior is mainly attributed to the stronger spectral overlap between red emission and the long-wavelength absorption region of AuNPs, which induces wavelength-selective optical coupling. By comparison, the PL spectrum of the green emitting layer is less affected by AuNPs, whereas that of the blue emitting layer is almost unchanged, which is consistent with the absorption spectral characteristics of AuNPs.

To quantitatively compare the PL decay dynamics, the normalized PL decay profiles in Figure 3b,d,f were fitted using a biexponential decay function, *I*(*t*) = *y*_0_ + *A*_1_ exp(−*t*/*τ*_1_) + *A*_2_ exp(−*t*/*τ*_2_), and the fitted profiles are overlaid as solid lines. The fitted lifetimes were used to compare the substrate-induced changes within each individual EML rather than to directly compare the intrinsic decay characteristics of different emitters.

For the red EML, the fitted lifetimes are *τ*_1_ = 2.65 μs and *τ*_2_ = 7.66 μs on PEDOT:PSS/EML(R). After incorporating AuNPs into PEDOT:PSS, the PEDOT:PSS + AuNPs/EML(R) sample shows comparable lifetimes of *τ*_1_ = 2.36 μs and *τ*_2_ = 6.91 μs, indicating that AuNPs embedded in PEDOT:PSS only slightly affect the PL decay dynamics of the red EML. In contrast, the AuNPs/EML(R) sample gives *τ*_1_ = 2.29 μs and *τ*_2_ = 16.81 μs. Although a longer fitted lifetime is obtained in the long-time region, the overall decay of AuNPs/EML(R) is clearly faster than that of PEDOT:PSS/EML(R), as also reflected by the reduced average decay lifetime of approximately 3.07 μs compared with 4.40 μs for PEDOT:PSS/EML(R) and 4.55 μs for PEDOT:PSS + AuNPs/EML(R). This accelerated decay, together with the red shift observed in the PL spectrum, indicates that direct contact or close proximity between the red EML and the AuNP film enhances exciton deactivation and introduces additional nonradiative quenching pathways.

For the green EML, the fitted lifetimes show only minor changes among the three substrates. The *τ*_1_ values are 0.378, 0.373, and 0.380 μs for PEDOT:PSS/EML(G), PEDOT:PSS + AuNPs/EML(G), and AuNPs/EML(G), respectively, while the corresponding *τ*_2_ values are 3.99, 3.95, and 4.09 μs. These close lifetime values indicate that AuNPs do not significantly modify the PL decay dynamics of the green EML. Similarly, for the blue EML, the fitted lifetimes are almost unchanged. The *τ*_1_ values are 0.364, 0.364, and 0.365 μs for PEDOT:PSS/EML(B), PEDOT:PSS + AuNPs/EML(B), and AuNPs/EML(B), respectively, and the corresponding *τ*_2_ values are 4.99, 5.04, and 4.97 μs. This confirms that AuNPs have negligible influence on the PL decay dynamics of the blue EML.

These results indicate that when AuNPs are in direct contact with the EML, their influence on the PL characteristics of the emitting layers is wavelength-selective, with the strongest effect observed for red emission, followed by green emission, and the weakest effect for blue emission. When AuNPs are embedded in PEDOT:PSS, the PEDOT:PSS matrix covers the AuNPs and increases the effective separation distance between AuNPs and emissive excitons in the EML, thereby weakening LSPR-mediated optical perturbation and exciton quenching. This result further supports the design rationale of this work: to exploit the electrical injection-modifying function of small-sized AuNPs in PEDOT:PSS while suppressing plasmonic coupling and nonradiative quenching of emissive excitons.

### 3.3. Influence of AuNP-Doped PEDOT:PSS on the Performance of OLEDs with Different Emission Wavelengths

The PL results discussed above demonstrate that the coverage of small-sized AuNPs by PEDOT:PSS can effectively weaken their optical perturbation to the emitting layer. Therefore, it is necessary to further evaluate the influence of AuNP-doped PEDOT:PSS as the hole injection layer on the electrical and electroluminescent characteristics of OLEDs.

OLEDs with three different emission wavelengths were fabricated by incorporating AuNPs at different concentrations into PEDOT:PSS, which served as the hole injection layer. The influence of small-sized AuNP doping on the electrical characteristics of the devices was investigated, and the correlation between this effect and the emission wavelength of the devices was further analyzed.

The structures of the AuNP-doped blue OLEDs were as follows:

ITO/PEDOT:PSS/EML (B)/DPEPO/TPBi/LiF/Al
ITO/PEDOT:PSS +1% AuNPs/EML (B)/DPEPO/TPBi/LiF/Al
ITO/PEDOT:PSS +5% AuNPs/EML (B)/DPEPO/TPBi/LiF/Al
ITO/PEDOT:PSS +10% AuNPs/EML (B)/DPEPO/TPBi/LiF/Al
ITO/PEDOT:PSS +20% AuNPs/EML (B)/DPEPO/TPBi/LiF/Al

**Figure 4:**
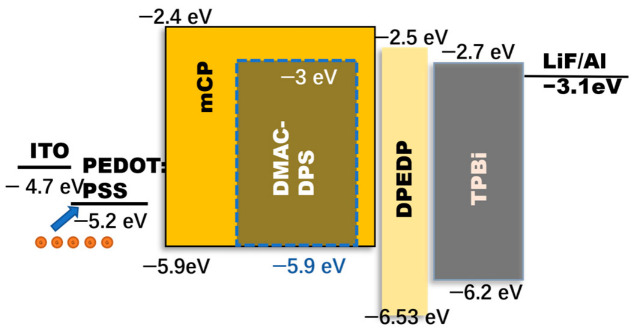
Energy-level structure of the blue OLEDs.

Figure 4 shows the energy-level structure of the blue OLEDs.

A single and stable blue emission band is observed in the EL spectra, and AuNP doping does not induce an obvious spectral shift. This can be attributed to two factors. First, the spectral overlap between AuNPs and blue emission is weak, making pronounced LSPR-mediated optical perturbation unlikely. Second, the AuNPs used in this work are small in size and embedded within PEDOT:PSS, which increases the effective separation distance between AuNPs and emissive excitons in the EML and thereby reduces their influence on surrounding emissive molecules. As a result, stable EL spectra are obtained.

**Figure 5:**
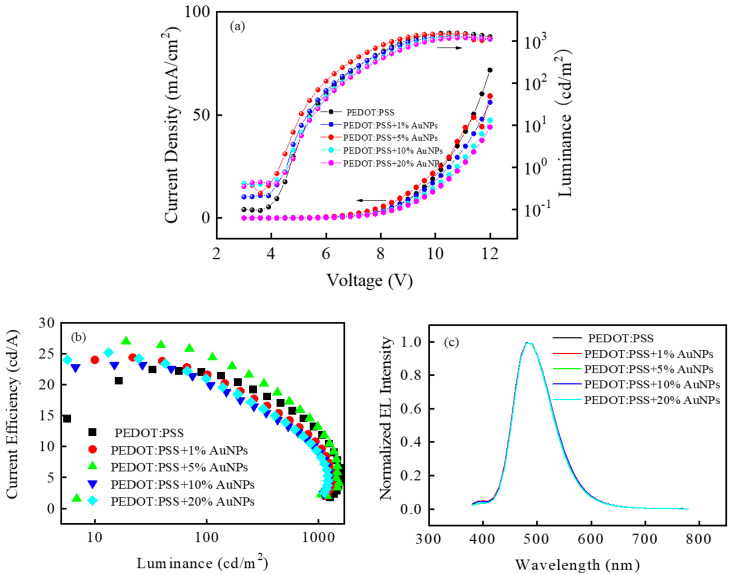
Device characteristics of blue OLEDs with different AuNP doping concentrations: (**a**) *J*–*V*–*L* curves; (**b**) current efficiency–luminance (CE–*L*) curves; and (**c**) EL spectra.

Figure 5a shows the *J*–*V*–*L* characteristics of the blue OLEDs. The *J*–*V*–*L* curves were obtained from stepwise forward voltage scans, and the plotted voltage range mainly covers the turn-on and electroluminescent operation region of the devices. With increasing AuNP doping ratio, the current density first increases and then decreases. Device 3 exhibits the highest current density and the lowest turn-on voltage. According to the energy-level structure, hole injection from PEDOT:PSS into the emitting layer needs to overcome an injection barrier of approximately 0.7 eV. Therefore, improving the hole-injection process is important for reducing the turn-on voltage and suppressing interfacial charge accumulation. When the AuNP doping ratio is increased to 5%, hole injection is enhanced, the turn-on voltage decreases from 4.8 V for the undoped device to 4.2 V, and an increased current density is observed. Figure 5b shows the current efficiency-luminance characteristics. The maximum current efficiency increases from 22.4 cd/A for the undoped Device 1 to 27 cd/A for Device 3 with 5% AuNP doping.

When the AuNP doping ratio is further increased to 10% and 20%, the device performance begins to decline relative to the optimum 5% device. This may be associated with AuNP aggregation in PEDOT:PSS at high doping concentrations, which can introduce additional hole-trapping sites or local defects and deteriorate the HIL/EML interfacial contact. Therefore, rational control over the size and doping concentration of AuNPs is essential for optimizing device performance.

Table 1 summarizes the performance of blue OLEDs with different AuNP doping ratios.

In addition, to further verify the applicability of AuNP-doped PEDOT:PSS to OLEDs with different emission wavelengths, red and green OLEDs were fabricated, and their device performances were investigated.

For red OLEDs, the phosphorescent material Ir(MDQ)_2_acac was selected as the red-emitting guest due to its strong emission capability. The device structure and fabrication conditions were established following mature solution-processed OLED protocols, including the host–guest composition and solution concentration. The structures of the AuNP-doped red OLEDs were as follows:

ITO/PEDOT:PSS/EML (R)/POT2T (40 nm)/LiF (1 nm)/Al (1000 nm)
ITO/PEDOT:PSS + 5% AuNPs/EML (R)/POT2T (40 nm)/LiF (1 nm)/Al (1000 nm)
ITO/PEDOT:PSS + 20% AuNPs/EML (R)/POT2T (40 nm)/LiF (1 nm)/Al (1000 nm)

**Figure 6:**
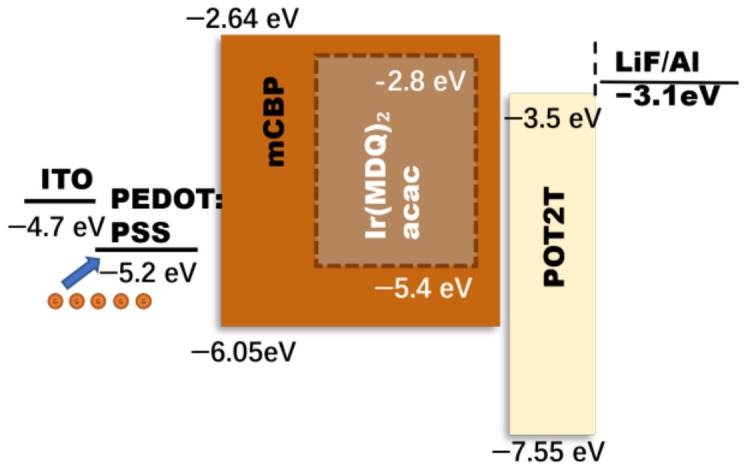
Energy-level structure of the red OLEDs.

Figure 6 shows the energy-level structure of the red OLEDs.

**Figure 7:**
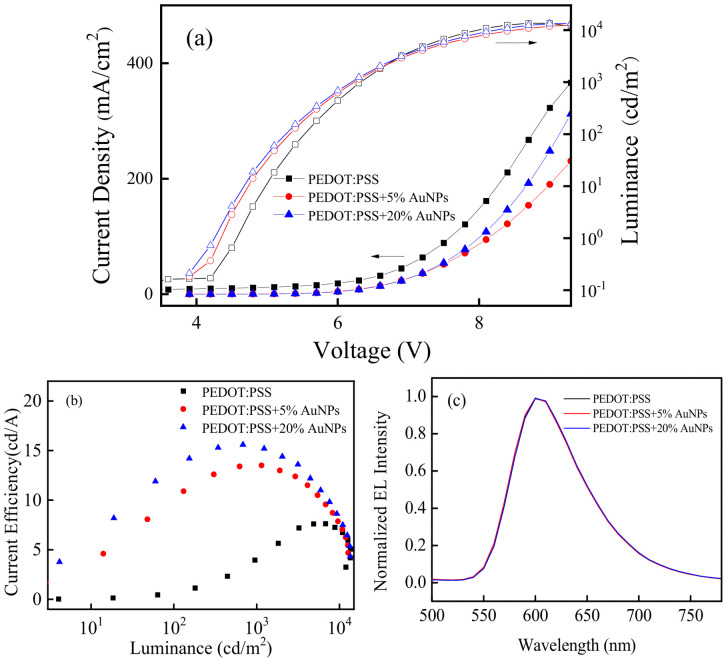
Device characteristics of red OLEDs with different AuNP doping concentrations: (**a**) *J*–*V*–*L* curves; (**b**) current efficiency–luminance (CE–*L*) curves; and (**c**) EL spectra.

Figure 7 presents the *J*–*V*–*L* characteristics, current efficiency–luminance (CE–*L*) curves, and electroluminescence (EL) spectra of red OLEDs fabricated using PEDOT:PSS doped with different concentrations of AuNPs. As shown in the *J*–*V*–*L* characteristics, the pristine PEDOT:PSS device shows a non-negligible current density below 5V despite its higher turn-on voltage, suggesting that the low-bias current is mainly associated with interfacial charge accumulation, trap-assisted transport, or leakage-type current rather than efficient radiative recombination. The AuNP-doped red devices exhibit lower current densities under both low- and high-voltage conditions. Among them, the device with 5% AuNP doping exhibits the lowest current density at high voltages, whereas the device with 20% AuNP doping shows a noticeable increase in current density in the high-voltage region. Based on the luminance and current efficiency results, both the 5% and 20% AuNP-doped devices exhibit lower turn-on voltages and significantly enhanced current efficiencies. The maximum current efficiency increases from 7.64 cd/A for the undoped device to 15.6 cd/A for the device with 20% AuNP doping.

The reduced turn-on voltage of the red devices indicates that AuNP-doped PEDOT:PSS facilitates hole injection. Notably, the current-efficiency enhancement is more pronounced in the red OLEDs, which may be partly associated with the larger hole-injection barrier at the PEDOT:PSS/mCBP-based red EML interface; in addition, the wavelength-dependent optical response of AuNPs in the long-wavelength region may also contribute to the enhanced red OLED performance.

Table 2 summarizes the performance parameters of red OLEDs with different AuNP doping concentrations.

For green OLEDs, 4CzIPN, a commonly used high-efficiency emissive molecule, was selected as the green-emitting guest. The fabrication procedure of the green OLEDs was also designed based on reported device structures, host–guest ratios, and solution concentrations. The structures of the AuNP-doped green OLEDs were as follows:

ITO/PEDOT:PSS/EML (G)/DPEPO/TPBi/LiF/Al
ITO/PEDOT:PSS + 5% AuNPs/EML (G)/DPEPO/TPBi/LiF/Al
ITO/PEDOT:PSS + 20% AuNPs/EML (G)/DPEPO/TPBi/LiF/Al

**Figure 8:**
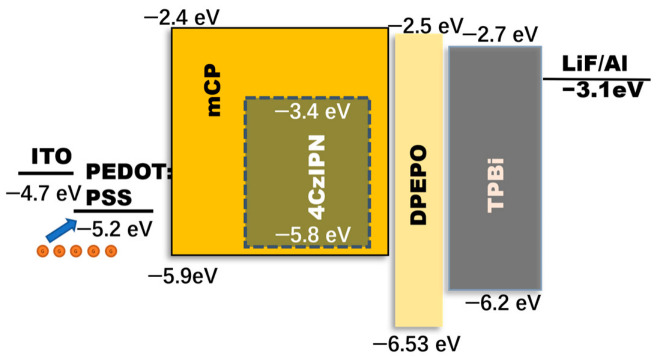
Energy-level structure of the green OLEDs.

Figure 8 shows the energy-level structure of the green OLEDs.

**Figure 9:**
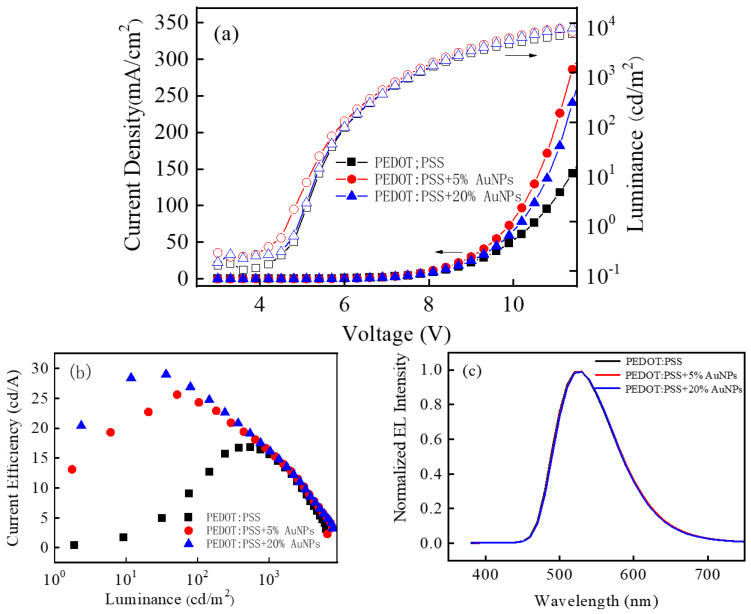
Device characteristics of green OLEDs with different AuNP doping concentrations: (**a**) *J*–*V*–*L* curves; (**b**) current efficiency–luminance (CE–*L*) curves; and (**c**) EL spectra.

Figure 9 shows the *J*–*V*–*L* characteristics, CE–*L* curves, and stable EL spectra of green OLEDs. Compared with the undoped device, the device with 5% AuNP doping exhibits a lower turn-on voltage, higher current density, and increased luminance at high voltages. The maximum current efficiency increases from 16.8 cd/A for the undoped device to 29.0 cd/A for the device with 20% AuNP doping. The variation in current density at low voltages, together with the reduced turn-on voltage, indicates that AuNP-doped PEDOT:PSS helps optimize hole injection and carrier balance within the device, thereby enhancing the current efficiency of green OLEDs.

Table 3 summarizes the performance parameters of green OLEDs with different AuNP doping concentrations.

### 3.4. Verification of Enhanced Hole Injection Induced by AuNP Doping in OLEDs

**Figure 10:**
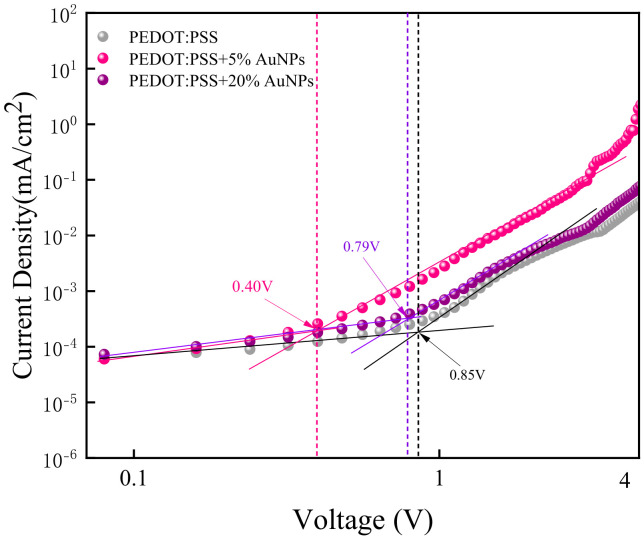
*J*−*V* characteristics of hole-only devices based on PEDOT:PSS and AuNP-doped PEDOT:PSS. The solid lines are linear fitting lines used to determine the characteristic transition voltages.

To further verify the influence of AuNP doping on the hole-injection process in OLEDs, hole-only devices with the structure of ITO/PEDOT:PSS + x%AuNPs/EML(B)/TCTA (30 nm)/MoO_3_ (10 nm)/Al were fabricated, and their *J*–*V* characteristics were measured. As shown in Figure 10, after doping with 5% and 20% AuNPs, the hole-only device exhibits a higher current density at the same voltage, indicating that AuNP doping helps improve the hole-injection and hole-transport capability of the PEDOT:PSS layer.

The *J*–*V* curves of hole-only devices can generally be divided into the ohmic transport region, the trap-filled limit region, and the trap-free space-charge-limited current region according to the slope variation in different voltage ranges. Among them, the trap-filled limit voltage, *V*_TFL_, is positively correlated with the trap-state density. Compared with the pristine PEDOT:PSS device, the AuNP-doped device exhibits a lower *V*_TFL_, indicating that a lower voltage is required to fill the trap states and that the trap-state density is reduced. Combined with the higher hole current density, this result suggests that AuNP doping optimizes the hole-transport process in the PEDOT:PSS layer and reduces trap-limited hole transport.

The trap-state density of the hole-only devices was estimated from the trap-filled limit voltage according to:

$$N_{t}=\frac{2\varepsilon_{r}\varepsilon_{0}V_{TFL}}{{eL}^{2}}$$

where *V_TFL_* is the trap-filled limit voltage, *ε_r_* is the relative dielectric constant, *ε*_0_ is the vacuum permittivity, *e* is the elementary charge, and *L* is the effective transport thickness. The hole-only device based on pristine PEDOT:PSS shows a *V_TFL_* of 0.85 V, after incorporating 5% AuNPs, *V_TFL_* decreases to 0.40 V. This result indicates that 5% AuNP doping effectively suppresses trap-limited hole transport and improves hole injection/transport. In contrast, the 20% AuNP-doped device shows a *V_TFL_* of 0.79 V, which is only slightly lower than that of pristine PEDOT:PSS. This implies that excessive AuNP incorporation may weaken the beneficial effect of AuNP doping, possibly due to AuNP aggregation or increased interfacial disorder.

**Figure 11:**
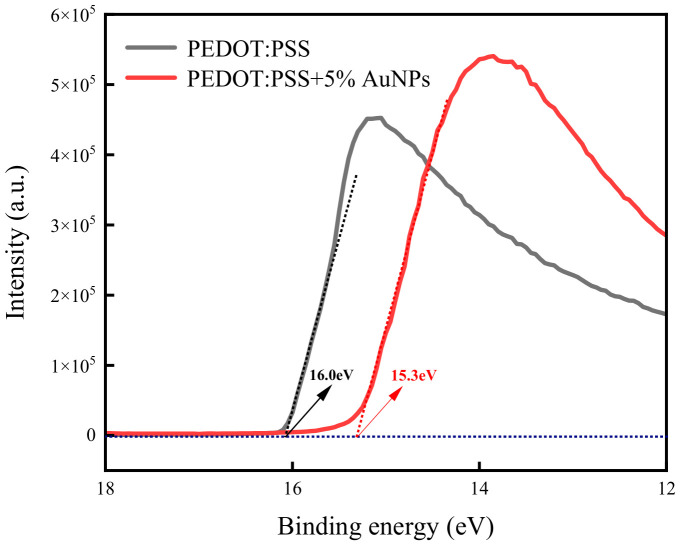
UPS secondary electron cutoff spectra of pristine PEDOT:PSS and PEDOT:PSS + 5% AuNPs films.

UPS measurements were further carried out to evaluate whether AuNP incorporation changes the interfacial electronic structure of PEDOT:PSS. As shown in Figure 11, the secondary electron cutoff shifts from 16.0 eV for pristine PEDOT:PSS to 15.3 eV for PEDOT:PSS + 5% AuNPs. According to Φ = h*ν* − E_cutoff_, where h*ν* = 21.22 eV for the He I excitation source, the work function increases from approximately 5.22 eV for pristine PEDOT:PSS to approximately 5.92 eV for PEDOT:PSS + 5% AuNPs. This increase in the effective work function indicates that AuNP incorporation modifies the surface electronic structure of PEDOT:PSS and makes its work function better aligned with the HOMO levels of the EML materials, thereby reducing the hole-injection barrier and facilitating hole injection from PEDOT:PSS into the EML.

In OLEDs, hole injection from PEDOT:PSS into the EML requires overcoming an interfacial injection barrier, which can lead to hole accumulation at the PEDOT:PSS/EML interface. This charge accumulation may disturb carrier balance and increase nonradiative recombination or exciton quenching. The hole-only device and UPS results demonstrate that AuNP doping enhances the hole-injection/transport capability of PEDOT:PSS by reducing trap-limited transport and increasing the effective work function. Therefore, AuNP-doped PEDOT:PSS can improve interfacial energy-level alignment, promote balanced carrier recombination, and contribute to enhanced OLED performance.

### 3.5. Electrical Impedance Spectroscopy of Devices Under Low-Voltage Charge Injection

Electrical impedance spectroscopy (EIS) measurements were carried out using an electrochemical workstation. For OLED solid-state devices, this measurement is mainly used to analyze charge injection, interfacial charge accumulation, and carrier recombination behavior under small-signal perturbation at different frequencies. At low voltages, carrier injection and transport in the organic functional layers are relatively weak, and the device mainly exhibits a pronounced capacitive response and high impedance. As the applied voltage increases, more carriers are injected into the device and the device gradually enters the conducting state. Under this condition, the impedance response can reflect carrier transport and recombination processes under operating conditions. To investigate the carrier transport behavior of the devices in the initial conducting state, a voltage of 5 V, corresponding to the onset of light emission in blue OLEDs, was selected as the DC bias. An AC perturbation signal of 5 mV was superimposed, and the frequency range was set from 100 kHz to 10 Hz. The blue OLED was selected as the representative device for EIS analysis because the blue-emission region has relatively weak spectral overlap with the absorption/LSPR response of AuNPs, which helps minimize wavelength-dependent LSPR coupling and exciton-quenching effects.

**Figure 12:**
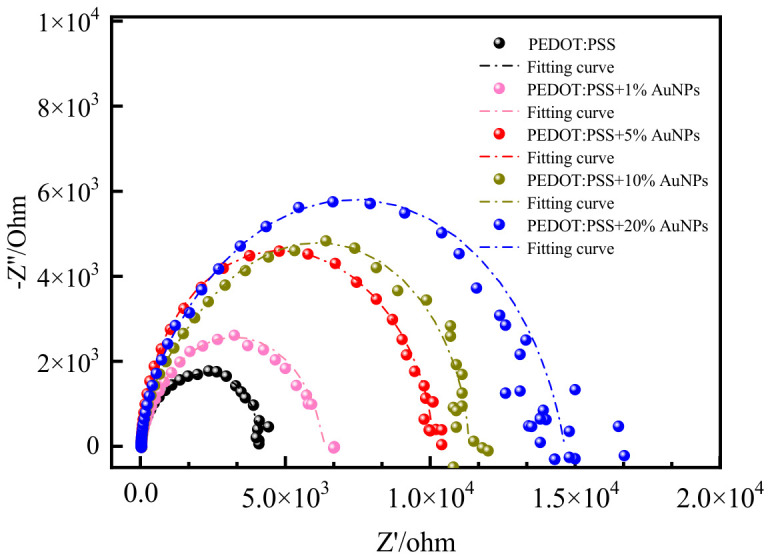
EIS Nyquist plots of blue OLEDs with different AuNP doping concentrations.

Figure 12 shows the EIS Nyquist plots of blue OLEDs with different AuNP doping concentrations. The semicircle radius of the AuNP-doped devices increases compared with that of the undoped device, indicating an increase in the equivalent impedance or recombination resistance of the devices in the initial conducting state. For blue OLEDs, holes injected from the ITO/PEDOT:PSS side and transported into the EML need to overcome a hole-injection barrier of approximately 0.7 eV at the PEDOT:PSS/EML interface, which can easily lead to interfacial charge accumulation. After AuNPs are doped into PEDOT:PSS, the hole-injection and interfacial carrier-transport processes are optimized, enabling more holes to enter the EML and recombine effectively with electrons, thereby reducing interfacial free-carrier accumulation and leakage-current contribution. The reduced accumulation of free carriers weakens the conductive response of the device to the AC small-signal perturbation, resulting in a larger semicircle radius in the Nyquist plots.

Therefore, the EIS results indicate that AuNP-doped PEDOT:PSS can regulate interfacial charge transport and recombination behavior in blue OLEDs under low-voltage initial conducting conditions, reduce interfacial charge accumulation, and, together with the hole-only device and *J*–*V*–*L* results discussed above, support the optimized hole-injection process induced by AuNP doping.

### 3.6. AC Voltage Response of OLEDs

**Figure 13:**
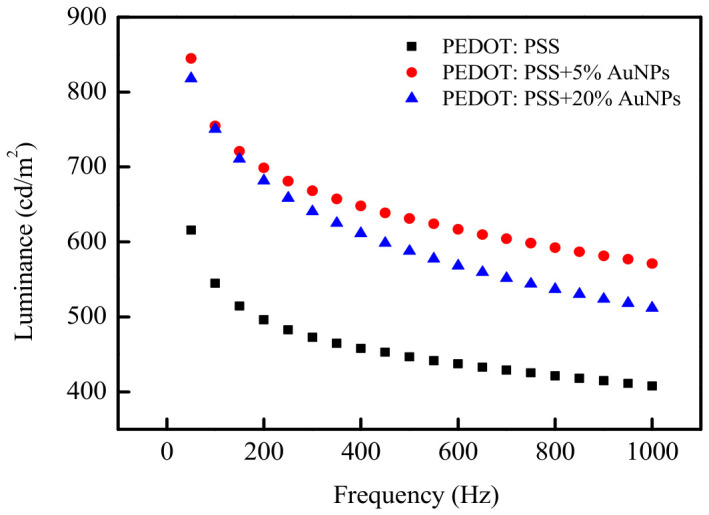
Luminance-frequency response curves of blue OLEDs under sinusoidal AC voltage.

To further verify the influence of AuNP doping on the dynamic charge injection and transport processes in the devices, the luminance-frequency responses of three blue OLEDs with different AuNP doping ratios were compared under sinusoidal AC voltage. In the test, the DC voltages corresponding to a luminance of 1000 cd/m^2^ were selected as the root-mean-square values of the applied AC voltage, which were 8.9 V, 8.5 V, and 9.6 V for the three devices, respectively. For a sinusoidal voltage, the peak-to-peak voltage is 2√2 times the root-mean-square value; therefore, the corresponding peak-to-peak voltages applied to the three devices were 25.2 V, 24.0 V, and 27.1 V, respectively. Figure 13 shows the luminance variation of the three blue OLEDs over the frequency range of 50–1000 Hz.

Under DC voltage, the three devices were adjusted to the same luminance of 1000 cd/m^2^, indicating comparable steady-state radiative recombination output. However, under AC voltage driving, the luminance response is closely related to the dynamic processes of carrier injection, transport, and recombination. For devices with weaker charge injection and transport capability, carriers cannot sufficiently complete injection, transport, and migration into the emissive recombination zone within each half-cycle under the alternating electric field. This reduces effective radiative recombination and leads to decreased AC luminance.

As shown in Figure 13, the luminance of all devices gradually decreases with increasing frequency, indicating that high-frequency AC driving limits effective carrier injection and recombination in the devices. Among the three devices, the undoped reference device exhibits the lowest luminance, with a luminance of approximately 615 cd/m^2^ at 50 Hz. In contrast, the AuNP-doped devices show higher luminance at the same frequency, and the device with 5% AuNP doping maintains higher luminance output over a relatively broad frequency range. These results indicate that after AuNP doping into PEDOT:PSS, the devices exhibit improved dynamic charge injection and transport capability under AC driving, and charge accumulation at the PEDOT:PSS/EML interface is alleviated, thereby contributing to enhanced device performance.

### 3.7. Influence of AuNP Doping on the HIL/EML Interfacial Contact

The performance of OLEDs is affected not only by the charge-injection process but also by the surface morphology of organic films and interfacial contact. Therefore, the surface morphology of the HIL after AuNP doping into PEDOT:PSS and its influence on the film formation quality of the EML were further analyzed.

**Figure 14:**
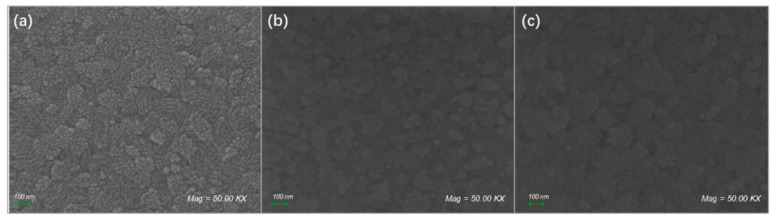
SEM images of surface morphology of PEDOT:PSS films doped with different AuNP concentrations, (**a**) for PEDOT:PSS without AuNPs; (**b**) for PEDOT:PSS doped with 5% AuNPs; (**c**) for PEDOT:PSS doped with 20% AuNPs.

Figure 14 shows the SEM surface morphologies of PEDOT:PSS films doped with different concentrations of AuNPs. The pristine PEDOT:PSS film exhibits densely distributed fine particle clusters with numerous small grooves on the surface. After doping with 5% AuNPs, the cluster size decreases, the grooves become shallower, and the film becomes more uniform and smoother overall. When the AuNP doping ratio is increased to 20%, larger-area aggregated features appear on the film surface, indicating that excessive AuNPs tend to aggregate in PEDOT:PSS. These results suggest that an appropriate amount of small-sized AuNPs helps fill the micro-grooves on the PEDOT:PSS surface and improve film flatness, whereas excessive doping increases the risk of aggregation.

**Figure 15:**
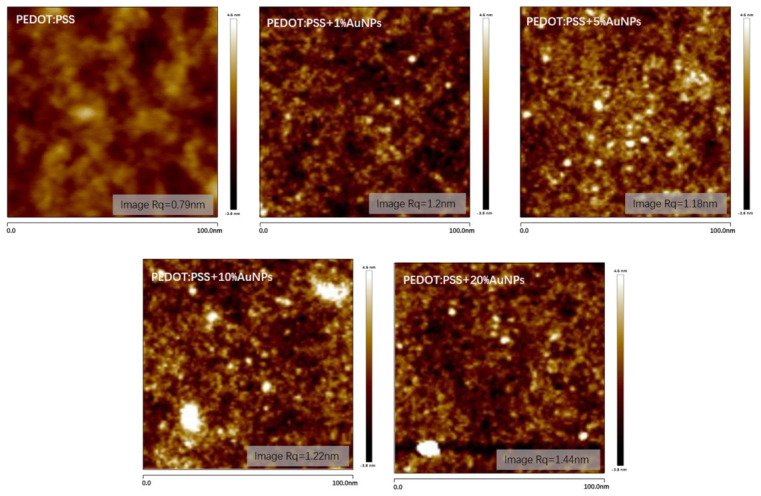
AFM images of PEDOT:PSS with different concentrations of AuNPs doped.

Figure 15 shows the AFM morphology images of PEDOT:PSS films with different AuNP doping ratios. As the AuNP doping ratio increases, both the number and size of bright spots in the films gradually increase. In the films doped with 1% and 5% AuNPs, the particle size is approximately 3–5 nm, which is consistent with the AuNP size observed by TEM, indicating that AuNPs can be well dispersed in the PEDOT:PSS film at low doping concentrations. In contrast, at doping ratios of 10% and 20%, aggregated particles with sizes of 10–15 nm appear in the films, suggesting that high AuNP concentrations reduce the morphological uniformity of the films. The AFM results also show that AuNP doping slightly increases the surface roughness of the films, with the roughness increasing from 0.79 nm for pristine PEDOT:PSS to approximately 1.2 nm for the film doped with 5% AuNPs.

**Figure 16:**
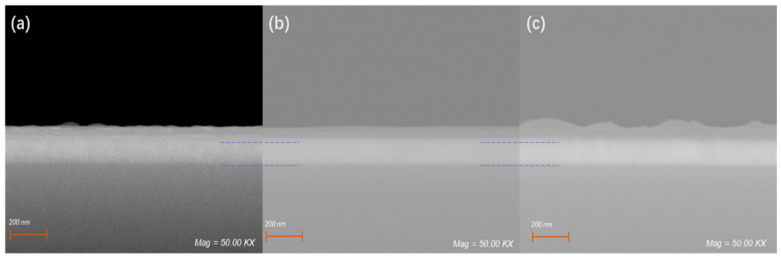
SEM cross-sections of multilayer films (ITO/PEDOT:PSS + x% AuNPs/EML), (**a**) for pure PEDOT:PSS; (**b**) for 5% AuNPs doped; (**c**) for 20% AuNPs doped.

Because solution-processed OLEDs undergo multiple spin-coating and annealing steps, the surface morphology of the HIL directly affects the subsequent film formation of the EML and the HIL/EML interfacial contact. To further examine the film formation of the EML on different HIL substrates, SEM cross-sectional images of ITO/(PEDOT:PSS):x% AuNPs/EML multilayer films were measured, as shown in Figure 16. In the cross-sectional images, the bottom layer is the glass substrate, the bright layer with a thickness of approximately 110 nm is the ITO layer, and the upper layers are PEDOT:PSS or AuNP-doped PEDOT:PSS followed by the EML. It can be observed that AuNP doping has little influence on the thickness of the PEDOT:PSS layer, whereas the surface morphology and flatness of the EML differ significantly. Compared with the EML deposited on pristine PEDOT:PSS, the EML deposited on PEDOT:PSS doped with 5% AuNPs exhibits a smoother surface, indicating that an appropriate amount of AuNP doping helps improve the HIL/EML interfacial contact. When the AuNP doping ratio is increased to 20%, the EML shows obvious surface fluctuations in the cross-sectional image and deteriorated film formation quality. This may be related to excessive AuNP aggregation and the difficulty in fully covering locally enlarged AuNP aggregates with PEDOT:PSS.

**Figure 17:**
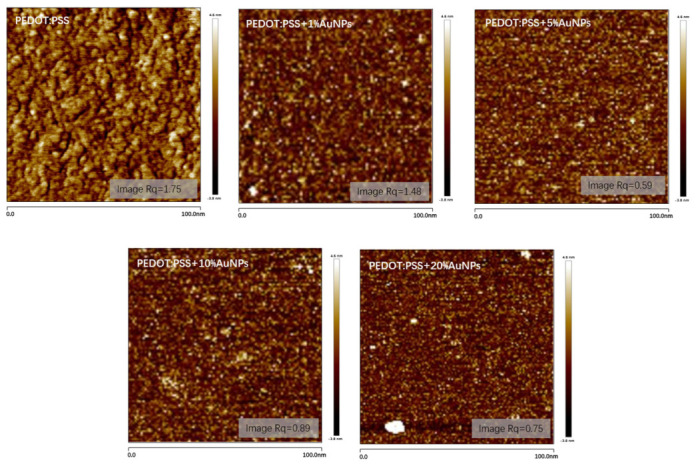
Phase images of PEDOT:PSS films doped with different concentrations of AuNPs.

Figure 17 further presents the phase images of PEDOT:PSS films doped with different concentrations of AuNPs. The phase roughness results show that after doping with 5% AuNPs, the phase roughness of the PEDOT:PSS film decreases from 1.75 nm for the pristine sample to 0.59 nm, indicating that an appropriate amount of small-sized AuNPs helps improve the phase-distribution uniformity of the PEDOT:PSS film. Because the AuNPs prepared in this work have small particle sizes and good dispersion, they can be effectively embedded in the PEDOT:PSS film at an appropriate doping concentration, thereby improving the uniformity of the surface structure. However, excessive AuNPs tend to aggregate into larger particles, which deteriorates film quality and interfacial contact.

The AFM roughness and phase roughness of PEDOT:PSS films with different AuNP doping ratios are summarized in Table 4.

Previous studies on the regulation of OLED performance by AuNPs have mainly focused on the LSPR effect. However, this effect is highly dependent on the emission wavelength, and related studies have therefore primarily concentrated on red and green devices. In contrast, this work emphasizes the role of small-sized AuNPs as electrical injection modifiers. The experimental results demonstrate that embedding small-sized AuNPs in PEDOT:PSS enhances hole injection, reduces the turn-on voltage, and improves current efficiency without introducing an additional blocking layer. Meanwhile, the PEDOT:PSS matrix increases the effective separation distance between AuNPs and emissive excitons in the EML, thereby effectively suppressing LSPR-mediated optical perturbation and exciton quenching. Therefore, small-sized AuNP-doped PEDOT:PSS improves the performance of red, green, and blue OLEDs, indicating that its primary function is not plasmonic emission enhancement but rather the improvement of hole injection and interfacial carrier balance.

## 4. Conclusions

In this work, small-sized AuNPs with diameters of 3.5–4.0 nm were synthesized and embedded in PEDOT:PSS to modify the hole injection layer of OLEDs. PL measurements revealed that exposed AuNPs induced wavelength-dependent optical perturbation, with the strongest influence on red emission and negligible influence on blue emission, whereas PEDOT:PSS-embedded AuNPs effectively suppressed LSPR-mediated AuNP–exciton coupling and exciton quenching by increasing the separation distance from the EML. When incorporated into PEDOT:PSS, AuNPs enhanced hole injection, reduced interfacial charge accumulation, and improved HIL/EML interfacial contact, leading to reduced turn-on voltages and increased current efficiencies for blue, red, and green OLEDs. The maximum current efficiencies increased from 22.4 to 27.0 cd/A for blue devices, from 7.64 to 15.6 cd/A for red devices, and from 16.8 to 29.0 cd/A for green devices. These results demonstrate that small-sized AuNPs embedded in PEDOT:PSS primarily function as electrical injection modifiers rather than plasmonic emission enhancers, providing a general strategy for improving OLED performance across different emission wavelengths while suppressing plasmonic quenching.

## Figures and Tables

**Table 1:** Electroluminescent characteristics of blue OLEDs.

HIL	*V*_on_ (V)	*L*_max_ (cd/m^2^)	CE_max_ (cd/A)
PEDOT:PSS	4.8	1568	22.4
PEDOT:PSS + 1% AuNPs	4.5	1318	24.4
PEDOT:PSS + 5% AuNPs	4.2	1528	27
PEDOT:PSS + 10% AuNPs	4.6	1213	23.2
PEDOT:PSS + 20% AuNPs	4.6	1208	25.2

**Table 2:** Electroluminescent characteristics of red OLEDs.

HIL	*V*_on_ (V)	*L*_max_ (cd/m^2^)	CE_max_ (cd/A)
PEDOT:PSS	4.8	13,582	7.64
PEDOT:PSS + 5% AuNPs	4.5	12,773	13.5
PEDOT:PSS + 20% AuNPs	4.5	13,310	15.6

**Table 3:** Electroluminescent characteristics of green OLEDs.

HIL	*V*_on_ (V)	*L*_max_ (cd/m^2^)	CE_max_ (cd/A)
PEDOT:PSS	5.1	6303	16.8
PEDOT:PSS + 5% AuNPs	4.7	7203	25.6
PEDOT:PSS + 20% AuNPs	5.1	7856	29

**Table 4:** Morphological and phase roughness values of PEDOT:PSS films doped with different concentrations of AuNPs.

	PEDOT:PSS	PEDOT:PSS + 1% AuNPs	PEDOT:PSS + 5% AuNPs	PEDOT:PSS + 10% AuNPs	PEDOT:PSS + 20% AuNPs
Morphological roughness (nm)	0.79	1.2	1.18	1.22	1.44
Phase roughness (nm)	1.75	1.48	0.59	0.89	0.75

## Data Availability

The original contributions presented in this study are included in the article. Further inquiries can be directed to the corresponding author.

## Abbreviations

The following abbreviations are used in this manuscript:

OLED	Organic Light-Emitting Diode
AuNPs	Gold nanoparticles
EIS	Electrical Impedance Spectroscopy
